# Antiseptic Dressing of Wounds in the Lower Animals

**Published:** 1892-03

**Authors:** R. W. Burke

**Affiliations:** Corresponding Member of the Italian Veterinary Academy, Army Veterinary Department, Ambala, India


### ANTISEPTIC DRESSING OF WOUNDS IN THE LOWER ANIMALS.

By R. W. Burke, F. R. C. V. S., Corresponding Member of the Italian Veterinary Academy, Army Veterinary Department, Ambala, India.

A good deal of apathy exists in regard to this important subject, both among veterinary surgeons and medical practitioners; some maintaining that it is useless, while others are of opinion that it is altogether impracticable in the case of the lower animals. These opinions are usually held by individuals who are unacquainted with the workings of the system called the "Antiseptic System." As in other questions so in this: it is absolutely necessary that one should be acquainted with the practical workings of a system before he attempts to pronounce judgment on its merits or demerits. It would appear that those who condemn as useless the antiseptic treatment of wounds in the lower animals, do so from a mistaken belief that to dress a wound antiseptically it is necessary to employ the carbolic spray. Nothing could be more erroneous. It may be as well to say that dressing wounds under the spray, or "Listerism," is only resorted to, by the practitioner of human surgery, in the case of a deep wound having excessive discharge. There are numerous other wounds, however, which are not very deep and attended with less discharge; and they may be better treated by a little more observance of the principles of antiseptic dressing as distinguished from "Listerism" *per se.*

The following questions naturally suggest themselves : What are the objects of antiseptic dressings? And what are the means at our disposal for attaining those objects? First, the objects of antiseptic dressing are primarily and chiefly these: (*a*) to prevent the death of tissue; (*b*) to favor the removal of dead tissue, and (*c*) to assist nature in attending to her wants in each case. All this may be effected in many different ways: we assist nature by giving *rest* to an injured part, by preventing *tension* in the wound, and in various other ways. Discharges, when excessive, are likely to accumulate and cause *tension* in a wound. A part that is in motion after being wounded is said to be in a state of *unrest.* Tension by pressure causes a breaking down of the healthy tissues, their disintegration and suppuration, or even mortification. A state of unrest prevents organization in a newly-formed blood-clot, or even assists degeneration and evacuation of it from the wound. The death of tissue is prevented by affording it rest, by relieving tension in it, and by removing dead tissue from it, because, as Sir James Paget has shown, "all newly-forming tissue has a tendency to become *like* the tissue near which or on which it lies." The means by which these desidereta may be attained are obvious and many. We give *rest* in a case of sprain of the back tendons by supporting the latter with firm bandaging, by raising the heel, or by creating surrounding swelling artificially by blister, as taught by John Hunter, etc.; in that of inflamed joint by transfixing the joint and preventing all motion in it; in that of inflamed eye by putting a shade over the eye and protecting it from the light; in the case of inflamed kidneys by the administration of diaphoretics; in inflamed bowel by stopping its peristalsis by the administration of sedatives; in injury to the brain by the administration of cathartics; in disease of the heart by cardiac sedatives; and so on. The means at our disposal, therefore, belong to the surgeon as well as to the physician.

Again, we relieve *tension* in a case of "bruised sole" by cutting away the bulging horn and allowing a free exit of the accumulated fluid found pent up between the horny sole and the solar aspect of the *os pedis*; in abscess by early lancing it; in hydrothoras by tapping; in deep-seated inflammation by the use of counter-irritants; and in deep-punctured or incised wounds, which are attended with much discharge, by drainage. There are various kinds of drainage which are employed, *viz.*, gutta-percha tube drainage; sponge drainage, and the capillary drainage. The first is employed when copious discharge is expected to result; the second, when drainage has to be effected over a large area, and where considerable discharge obtains; while capillary drainage is used chiefly in the case of punctured and deep wounds, where it is intended to convey the discharge upward to the surface of the wound. The materials employed in capillary drainage are cat-gut and horse-hair. Several plaits of these are cut and secured in position in a wound where drainage is required. Some prefer the cat-gut, others the horse-hair. They both act on the principle of suction, which is carried on through the spaces present between the two strands of the cat-gut or horse-hair used. The sponge acts on a similar principle, and there is no better sucking agent that can be employed. The cat-gut, horse-hair or sponge is only used in the case of extensive wounds; it does not spoil after usage, and may be utilized for several cases, if properly cleaned and disinfected every time it is used. The gutta-percha tubes are of various diameters, being perforated throughout their whole extent longitudinally, and here and there latterally. They are only necessary where active disintegration of tissue and copious discharge are present.

We thus assist nature in many and varied ways in guarding against the danger of putrefaction and death of tissue, and so claim to treat wounds, in the phraseology of the present day, antiseptically. We try to prevent the germs of putrefaction from getting a suitable pabulum wherein to lodge and spread their destructive influence. And, as it has been shown, this desirable end is attained in ways apart from Listerism properly so-called. Listerism, or the treatment under the carbolic spray, is necessary only in certain cases. What are those cases where it is indicated, and those where it is not required? It is necessary (1) in injuries to flat bones; (2) in injuries to serous membranes accidentally exposed to the external air, as in "opened joint," in wounds of the chest, as in broken ribs; (3) in injuries to fibrous membranes, as the periosteum, sheaths of tendons, and fasciæ of muscles; (4) in deep punctured and incised wounds; (5) where profuse discharge is present, and (6) septicæmia is threatened, and (7) when there is considerable loss of tissue and a large blood-clot has to be organized. It is not indicated where all these conditions are absent, and when the wound is superficial and its base callous.

One desideratum in the antiseptic system of treatment of wounds is to prevent death of tissue. Lister does this by preventing the access into a wound of germs which are engaged in causing the destruction of all nearly injured structures. This method of prevention of death of tissue is known under the name of "Listerism." Listerism is the most perfected part of the Antiseptic System, though it is not the whole of it. It consists of a "deep" and a "top" dressing, which are employed always under the carbolic spray. The deep dressing may not be necessary in all cases, such as small wounds and when there is little discharge present. Folds of gauze and sponges are used as absorbants in the "top" dressing when much discharge is expected; while cat-gut, horse-hair, or gutta-percha tnbes are used in the "deep" dressing as a drainage and with a similar object. Immediately over the wound is placed the "green-protective," which is a kind of oil-cloth intended to afford protection to the injured surface of the wound against the materials employed in the dressing. It also scts as a kind of diffuser of the discharges that result from the wound, and thus obviates *tension* in it by preventing the accumulation of fluids. Over this protective is used the boracic lint, previously dipped in carbolic solution. Over the "top" dressing, and where much discharge is suspected, is placed the Mackintosh cloth, which is also a kind of oil-cloth, and intended to promote diffusion of the discharges brought to the surface by the different modes of drainage employed. Over all the bandage is applied, which is secured in position by the use of safety pins. All materials used in dressing, as well as the various appliances, the surgical instruments and the surgeon's hands, should be previously dipped in a solution of carbolic acid (1 to 40). No part of the dressing should be attempted except under the carbolic spray.

*Carbolic spray.*—There is a special apparatus brought into requisition in the use of the carbolic spray. By applying heat to a chamber in the apparatus containing a 1 to 40 solution of carbolic acid, the latter is given off in the form of a vapor, which rises and gains exit through a minute orifice in a tubular projection attached to the top of the apparatus, which is termed the "antiseptic spray producer." Various patterns of the "antiseptic spray-producer" are now in use,—improvements in construction being constantly suggested by different surgeons.

Listerism has scored an appreciable triumph over all older methods of treatment in operation. (1) French surgeons try the cold water treatment of wounds; this amounts, in fact, to removal of dead tissue from a wound, alluded to on a former page. (2) Some surgeons try the treatment under a scab, by painting a wound with collodion, &c., and making an artificial scab; but, in our opinion, this is not good practice, inasmuch as the effusion under the scab is liable to collect and cause tension, which will favor degeneration and slough of the newly-formed blood-clot in the wound. (3) Native practitioners try *neem* fomentation of wounds; this favors natural drainage, as well as surrounds the wound with a germicide vapor; but when a large clot of blood is undergoing organization it is liable to favor its degeneration and ejection. (4) The treatment of opened-joint by blister, with the view of closing up the orifice and preventing access of air into the joint, is also an antiseptic method of treatment. For old standing wounds and ulcers having a putrescent discharge, a callous base, and being slow to heal, chloride of zinc, iodoform, and lead ointment are employed; they promote the healing process by acting both as antiseptics and astringents.

What are the benefits resulting from a due observance of all the principles of Antiseptic treatment? They are these: (1) No putrefactive irritation; (2) no death of tissue; (3) no pain resulting from inflammatory tension; (4) no putrefactive poisoning, or septicæmia, due to absorption of putrid material from the wound; (5) no spreading gangrene; (6) diminished period during which the animal must be kept from work. The principles of antiseptic dressing displace evil by crowding it out from the good, which is done in accordance with the law of Paget—"all tissue has a tendency to become like the tissue near which or upon which it lies."

That these benefits are real, and not imaginary, is attested by the results in practice. It has been stated by some, in regard to septicæmia as a result of putrefaction in the local sore, that the lower animals are not subject to attacks of this disease. Nothing could be more erroneous. It is not matter of opinion, but something which the experienced surgeon can easily determine for himself in doubtful cases. It is well known that following the infliction of some wounds, whether in man or in the lower animals, fever sets in. It is contended by some veterinary surgeons that the fever attending such wounds is only the result of pain, or what is called traumatic or "irritative fever." In some cases this may be true; in others it is not. Fever resulting from pain, as well as fever which is induced by absorption of aseptic tissue detritus, is characterized by totally different symptoms, by totally different results from those peculiar to the septic disease. It will be sufficient to state here, that the clinical thermometer should always be employed in all cases of doubt and where the diagnosis is uncertain.

What are the disadvantages of the Antiseptic system? The aim of the system being to guard against putrefaction, substances are employed called antiseptics, which have the power of preventing sepsis. All workable antiseptics, so far as we yet know, are *irritants*; so that, in all probability, they act by destroying organisms which are the cause of putrefaction. Thus, when carbolic acid, boracic acid and salicylic acid are applied to the tissues, they cause irritation.

What shall we say of carbolic liniment, of the strength of one to sixteen and one to eight, recommended by the older practitioners? This method of treatment is quite right, but only in superficial wonnds; whereas in deep-seated wounds, where the slightest excitement on the part of the animal at the time of dressing will give rise to absorption of air, loaded with septic germs, through the expansion of the cellular structures composing the walls of the wound, it will be of little use. In newly injured tissues and in healing ulcers the carbolic liniment will certainly prove injurious, as it is an irratant and causes a breaking down of the blood-clot and destruction of the granulation tissue.

How often should the dressings be changed in Listerism? This will depend upon the indications presented by individual cases. When the discharge has soaked through the top dressing, or when the Mackintosh is stained by putrefactive changes, the dressing should be renewed. This may not be necessary for a whole week in many cases. It is only necessary to undo the bandage and examine the Mackintosh, to see whether the dressing requires renewal or not. If the Mackintosh is clean and dry and there is no evidence of the discharge having reached the surface, the dressing must not be further disturbed, and the bandage again applied and secured in place with safety pins. It does not matter whether this is done under the carbolic spray or not. But the "deep" dressing must never be disturbed except under the spray.

When and in what class of cases is Listerism admissible? It is admissible in all cases where the time of receipt of injury does not average more than 12 to 24 hours, and where putrefactive change has not already set in. It is also admissible where a putrid sore is convertible into a non-putrid one. Thus, in comparatively superficial wounds, and where there is no communication of the air with serous cavities, as exist in opened-joint, in injuries to the thorax, etc., a septic wound may be made into an aseptic one by judicious cleansing of the wound and removal of the septic products from it by thorough washing. If, however, the septic changes are pronounced, and the subjacent tissues are infiltrated with the products of septic decomposition, this latter mode of dressing will be impracticable. No wound is convertible from a septic into an aseptic state, except where the products of decomposition are slight in amount and are removable, and where the deeper tissues are not infiltrated to any extent or otherwise implicated in the septic process. Experience will indicate the convertible cases. The efficacy of carbolic acid in the treatment of chronic ulcers is much inferior to many other applications. Several such are in use, but especially the boracic acid ointment made after Lister's formula, of one part white wax, one part boracic acid, two parts paraffin, and two parts almond oil, modified and softened by a little glycerine. Mr. Hutchinson's "antiphlogistic lotion," made of acetate of lead two parts, acetic acid one part, rectified spirit 16 parts, is also very useful in slowly healing, irritable ulcers. The acetate of lead and sulphate of zinc lotion recommended by Professor Dick, of the Edinburgh Veterinary School, is also useful in similar cases. Some American surgeons speak highly of the carbonate of lead paste, and we have found it beneficial in chronic ulcers with slight discharge; while chloral lotion and iodoform powder will be especially useful in cleansing dirty ulcers. Lotio ferri will be found highly advantageous in pale anæmic ulcers with watery discharge. We must shun the overuse of wet applications to chronic ulcers, and the surrounding skin should be kept as dry as possible. In a healthy sore, where cicatrisation is required, the lotion most rapid in producing the desired effect is chloride of zinc. The cautery has been found useful in phagedenic ulcers. Myrrh, alum and sulphate of iron powder answer well in indolent ulcers; tincture of myrrh and aloes in similar cases.

We prefer to think that Veterinarians have modified their views with regard to the difficulties said to be inseparable from an easy application of the antiseptic system to the treatment of wounds in the lower animals; further, that the above regime will not be treated with the former indifference. Almost everyone is ready to confess himself an expert in the healing art, but to numerate individual principles is a position few are willing to take. It is but too common to take refuge behind redundancy of knowledge; but to analize that redundant knowledge and see where its benefits lie, presumes a success which experience alone begets.

My main object in appealing to the above principles, is to refute the many strange opinions that are prevalent in regard to the application of the antiseptic system of treatment in the lower animals. The ordinary and plausible answer to the statement that this system of treatment is not applicable in the case of the lower animals, is that, however theoretically sound it may be, it is in practice remedied. For, as will have been seen from the foregoing description, the antiseptic system does not include Listerism only. It is said that we are crude to the extreme in our mode of dressing wounds in the lower animals. Perhaps I may convince some of my readers that, if we are crude, we are not without the wish to improve upon our past crudeness.

To summarize : What are the chief points to be borne in mind in the treatment of wounds? They are : (*a*) to stop the bleeding on the receipt of injury ; (*b*) to remove foreign bodies which act as irritants ; (*c*) to assist drainage ; (*d*) to avoid putrefaction ; and (*e*) to advise special modes of treatment in special cases.

(*a*) The first is done by applying ice to the bleeding part ; by the use of styptics, plugs, ligatures, etc.

(*b*) *Drainage.*—Without effecting drainage, healing by primary union cannot be properly established. If the wound be stitched up from end to end, the effusion collects, giving rise to *tension*, which causes irritation, inflammation and suppuration. The drainage tube has certain disadvantages unless antiseptic precautions are used. Putrefactive organisms may pass up the cavity of the tube and give rise to putrefaction. Secondly, in consequence of its elasticity, the india-rubber tube is apt to be compressed. And lastly all varieties of drainage (india-rubber) tubing contain more or less sulphur; hence sulphate of lead is formed and the green protective is stained, and it is difficult to tell whether this staining is due to putrefaction or to the drainage tube.

Sponge is employed when much effusion or hæmorrhage is expected, to act as capillaries. Gauze and catgut are also used with a similar object. Mr. Chiene, of Edinburgh, introduced the use of horse-hair drainage. Horse-hair acts like catgut, by capillarity. A sponge, when dry, consists of a mass of capillary tubes. Thus, a dry sponge placed externally on a wound helps, by capillary attraction, the flow of the discharges, and prevents tension, irritation, suppuration, and destruction of the newly-formed blood-clot in it.

(*c*) *Foreign bodies.*—These act as irritants in a wound. Hence the necessity of thorough cleansing of the wound prior to all dressing. This will be necessary in all cases. For even when a wound is carefully dressed according to antiseptic methods, and the discharge is non-putrid, yet it may be *dead* and constitute "waste-matter," which, if not washed out, will be likely to be absorbed by the lymphatics of the part and introduced into the general circulation, occasioning a rise in temperature or fever. The causes of rise in temperature, as considered by the surgeon, are three, viz., (*a*) pain; (*b*) absorption of refuse matter; (*c*) absorption of putrid matter.

(*d*) *To avoid putrefaction.*—The most perfect means of doing this is the science of "Listerism," or the practice of dressing wounds according to Lister's method, which has been described on a preceding page.

(*e*) *Special treatment.*—If union by granulation be desired the wound is generally stuffed, so as to prevent the edges from growing together by primary union. If union by scabbing be desired, the best agent for the formation of an aseptic scab is asceptic lint; this method is practicable only in the case of small wounds and where debility is not a prominent symptom. Should any tension under the scab occur, the part must be immediately relieved by puncturing the scab. As a rule, the treatment under a scab answers best in canine surgery. In dogs a great deal of absorption goes on in wounds, which is less perceptibly present in other patients. Hence, wounds in dogs are attended with less danger of tension, which is not the case in all animals. Dogs, moreover, lick their own sores, and a natural scab is formed which protects the wound.

In large, incised wounds, stiches will be necessary. In contused wounds, stitches are not indicated, as the vitality of the wound is lowered and stitches tend to increase this condition.

In the treatment of punctured wounds the great thing to be borne in mind is efficient drainage; the depth bearing no relation to the size of the external opening, the drain must lead from the bottom of the wound to the external opening.—*The Veterinary Journal.*

